# Obtaining Poly(3-Hexylthiophene) (P3HT) by Electropolymerization as an Alternative for the Substitution of Initiators with Free Radicals

**DOI:** 10.3390/polym17192656

**Published:** 2025-09-30

**Authors:** Christopher Uriel Landa Valdivia, Román Cabrera Sierra, Jesús Israel Guzmán Castañeda, Karla Jenny Lozano Rojas, José Antonio Barraza Madrigal

**Affiliations:** Escuela Superior de Ingeniería Química e Industrias Extractivas, Instituto Politécnico Nacional (ESIQIE—IPN), Av. Luis Enrique Erro S/N, Unidad Profesional Adolfo López Mateos, Zacatenco, Gustavo A. Madero, Ciudad de Mexico C.P. 07738, Mexico; landac315@gmail.com (C.U.L.V.); rcabreras@ipn.mx (R.C.S.); j.israelgc@yahoo.com.mx (J.I.G.C.); klozanor@ipn.mx (K.J.L.R.)

**Keywords:** P3HT, electropolymerization, FT-IR, semiconductor polymers, free radicals

## Abstract

In this work the production of poly(3-hexylthiophene) (P3HT) by electropolymerization using different materials as working electrodes is reported. Initially, the tests were carried out under atmospheric conditions with all the electrodes, and subsequently those that showed the best performance were selected to repeat the experiments in an inert atmosphere. The formation of the polymer film on the electrode surface was characterized by Fourier transform infrared spectroscopy (FT-IR) in the mid-infrared region (4000–400 cm^−1^). This technique allowed the evaluation of the transmittance of P3HT deposited on the electrode surface. The presence of the polymer was confirmed by the appearance of characteristic absorption bands at 2920 cm^−1^, 2850 cm^−1^, 850 cm^−1^ and 730 cm^−1^. The absorption peaks found at 2920 cm^−1^, 2850 cm^−1^ and 850 cm^−1^ show the presence of the typical functional groups of P3HT. These results suggest that the proposed method could represent a viable alternative for obtaining semiconductor polymers, avoiding the use of initiators with free radicals potentially harmful to human health.

## 1. Introduction

In the polymer industry, the use of free radical initiators remains a common practice in the polymer synthesis, primarily due to their low cost and effectiveness. These initiators break the double bonds of the monomers, leading to polymer chain growth [1,2]. Studies have indicated that these compounds release harmful agents with adverse effects on both the environment and human health, causing cellular aging and the potential development of diseases such as cancer [3,4,5]. Over time, new techniques have been suggested for the synthesis of sustainable and environmentally friendly semiconductor polymers, such as direct arylation, chemical oxidation and coupling polymerization methods [6,7,8,9].

Other methods include irradiation, such as gamma rays or electron beams, to generate free radicals and promote polymerization; as well as olefin metathesis polymerization, which uses olefin metathesis catalysts [10,11].

Semiconductor polymers, also known as intrinsically conducting organic polymers (ICPs), are made up of an insulating polymer in which a conductive phase, usually composed of metals or carbon powder, is dispersed [1]. Examples of these polymers are: poly(p-phenylene) (PPP), polyacetylene (PA), polythiophene (PT), poly(p-phenylene-vinylene) (PPV), polyaniline (PANI) and polypyrrole (PPy) [3,12,13].

Among these, polythiophene (PT) is a semiconducting polymer with unique electrical behavior, excellent thermal stability, and magnetic and optical properties [14]. However, its main disadvantage lies in its poor solubility in most organic and inorganic solvents due to its strong interchain interactions, which makes difficult its processing [6].

This study addresses the case of poly(3-hexylthiophene) (P3HT), a polythiophene (PT) derivative that is part of the poly(3-alkylthiophenes) (P3ATs or PATs) [6,15]. The presence of its alkyl side chain, this polymer has good solubility in organic solvents, which facilitates its processing in the form of thin films or coatings on substrates, a desirable characteristic for its integration into electronic devices [15,16,17,18]. P3HT is a p-type semiconductor, making it suitable for use in organic solar cells and organic transistors. It is distinguished by its high electronic conductivity, nonlinear optical properties and its ability to emit light by electroluminescence. Therefore, it has been widely used in electronic devices and in the manufacture of field effect transmitters (FETs) and organic light-emitting diodes (OLEDs), demonstrating its high stability and durability [7,19,20,21,22,23].

Semiconductor polymers can be obtained by electrochemical polymerization, applying a potential capable of inducing the oxidation of the monomer. This oxidation promotes the breaking of the double bonds, allowing the molecules to react with each other and link together, resulting in the formation of the polymer. This mechanism is comparable to that observed in chemical oxidation polymerization due to the formation of dimers, trimers and long chains [1,2,3]. Electropolymerization is usually performed using cyclic voltammetry, a versatile electrochemical technique for the study of electroactive species.

There are variables to consider when performing electropolymerization, which are: pH control, temperature [24], solvent, applied potential, reaction time, monomer concentration, material and size of the working electrode, since some materials are more prone to film formation on their surface [25,26,27,28,29].

For electropolymerization, it is essential that the electric current flows through the monomer solution, using a supporting electrolyte that allows ionic conduction in the medium, such as an ionic liquid (ILs), which are organic salts formed by ionic pairs composed of a cation and an anion of different natures. Also known as “green solvents”, ILs have several advantages, such as their intrinsic properties, low toxicity, flammability, good ionic conductivity, and chemical and thermal stability. In addition, they have the ability to dissolve organic and inorganic compounds in a temperature range close to ambient, which has been of great interest in various research areas [30]. This work proposes the production of poly(3-hexylthiophene) (P3HT) by electropolymerization as an alternative to the conventional use of free radical initiators in the production of semiconductor polymers. To achieve this, lithium hexafluorophosphate (LiPF6) was used as a supporting electrolyte, commonly used as ionic liquid in rechargeable batteries. This approach is more sustainable and safer, as it eliminates agents potentially toxic to human health and reduces the environmental impact of the process.

## 2. Materials and Methods

### 2.1. Working Electrodes

Copper, zinc, tin, 316 stainless steel, aluminum, vitreous carbon, nickel, fluorine-doped tin oxide (FTO), gold and platinum were used as working electrodes. Most of these electrodes were mechanically polished using different silicon carbide papers until a uniform surface and then chemically cleaned to remove surface impurities.

### 2.2. Solvent and Monomer Solution

A 0.01 M solution of lithium hexafluorophosphate (LiPF6) and 0.01 M 3-hexylthiophene (3HT) was prepared in a volume of 50 mL. A solvent solution was prepared with acetonitrile (AcN) and tetrahydrofuran (THF) by mixing 45 mL of AcN and 5 mL of THF at room temperature, followed by the addition of 76 mg of LiPF6 and 90 μL of 3HT, thus achieving the desired concentrations of both compounds.

### 2.3. Tests Under Atmospheric Conditions and in an Inert Atmosphere

To identify the appropriate materials for polymer formation, tests were performed using different working electrodes under atmospheric conditions. Thereafter, some electrodes were selected to evaluate the feasibility of producing a semiconductor polymer under an inert atmosphere. To achieve this, the solution was bubbled with nitrogen gas for 10 min before experimentation and maintained throughout the tests.

### 2.4. Electropolymerization

**Figure 1:**
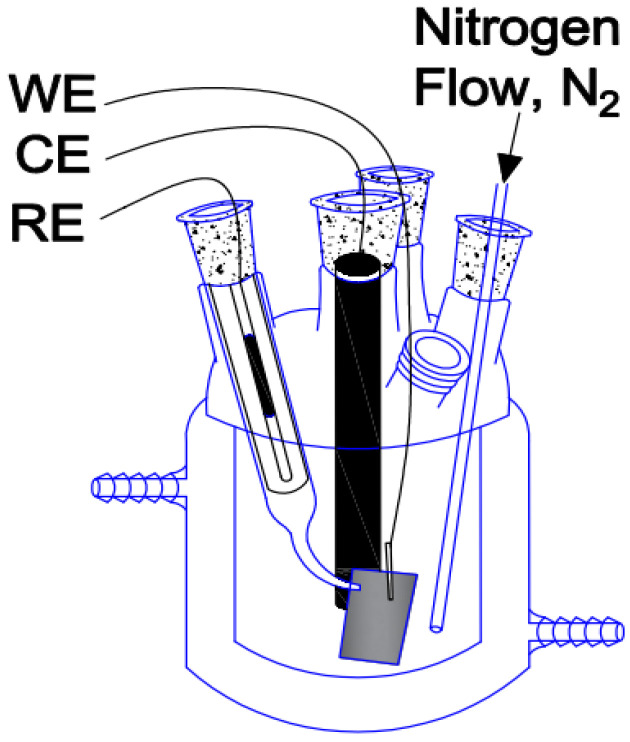
Electrochemical cell layout: working electrode (WE), counter electrode (CE), reference electrode (RE), nitrogen flow (N_2_).

The electropolymerization was carried out in a typical electrochemical cell, using the previously prepared working electrodes, a graphite electrode (counter electrode) and a silver/silver chloride (Ag/AgCl) as reference electrode (Figure 1). The voltammetry measurements were performed in a potential range from −0.5 to 1.5 V from the open circuit potential and scan rate of 50 mV/s, using a BioLogic SP-300 potentiostat/galvanostat.

**Figure 2:**
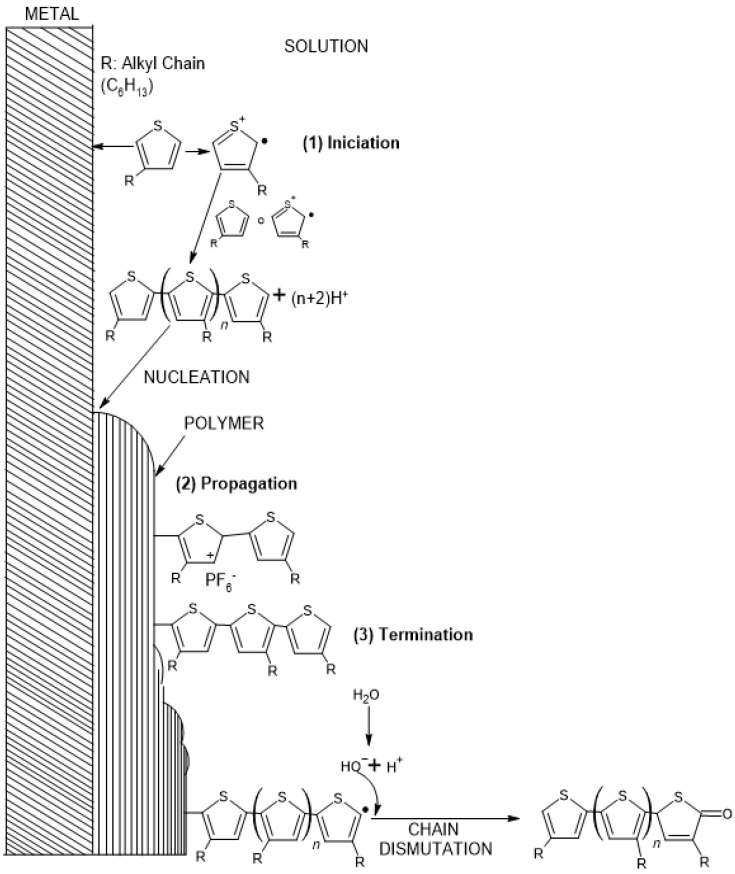
Stages of electropolymerization at working electrode.

Figure 2 shows the stages of the electropolymerization process at the electrode surface. The initiation stage (1) involves the formation of the first free radical or “seed”, which, through resonance, seeks the most stable position in the molecule. The stages are inspired in the model proposed in [3]. The propagation stage (2) involves the growth of the polymer chain on the electrode surface, generating overlapping layers. Finally, the termination stage (3) can be generated in two ways: by joining two chains or by dismutation termination, in which the end of the polymer chain has a carbonyl group due to overoxidation of the material.

**Figure 3:**
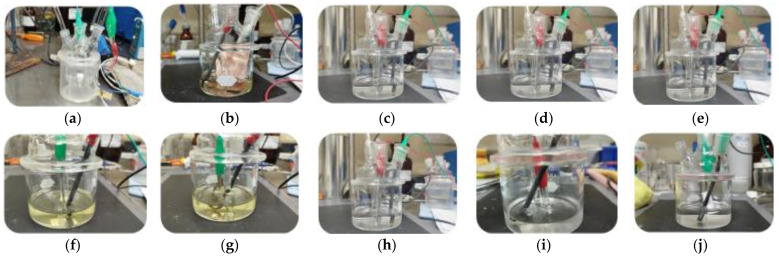
Tests under atmospheric conditions; (**a**) Test with 316 stainless steel electrode and platinum counter electrode; (**b**) Test with copper electrode and graphite counter electrode; (**c**) Test with zinc electrode and graphite counter electrode; (**d**) Test with tin electrode and graphite counter electrode; (**e**) Test with aluminum electrode and graphite counter electrode; (**f**) Test with nickel electrode and graphite counter electrode; (**g**) Test with gold electrode and graphite counter electrode; (**h**) Test with platinum electrode and graphite counter electrode; (**i**) Test with FTO electrode and graphite counter electrode; (**j**) Test with glassy carbon electrode and graphite counter electrode.

**Figure 4:**
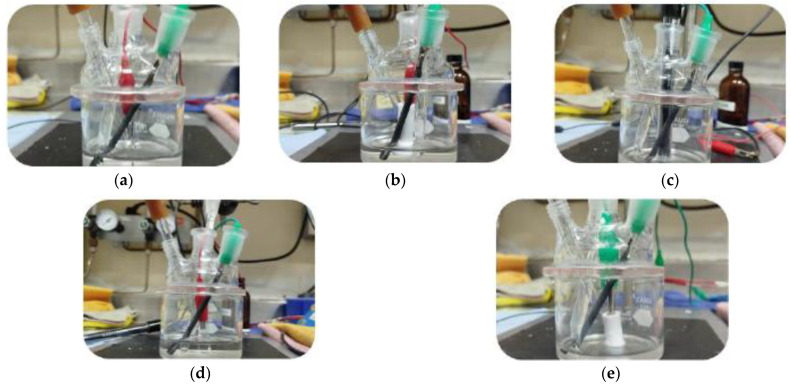
Tests in an inert atmosphere; (**a**) Test with nickel electrode and graphite counter electrode; (**b**) Test with gold electrode and graphite counter electrode; (**c**) Test with platinum electrode and graphite counter electrode; (**d**) Test with FTO electrode and graphite counter electrode; (**e**) Test with glassy carbon electrode and graphite counter electrode.

Figure 3 and Figure 4 show the tests under atmospheric and inert conditions, respectively. In some cases, a color change was observed in the solution, while in others, a translucent hue was observed.

### 2.5. Fourier Transform Infrared Spectroscopy

The resulting polymer was characterized by Fourier transform infrared spectroscopy (FT-IR), using a 65 FT-IR spectrophotometer Perkin Elmer equipped with an attenuated total reflectance (ATR) accessory. Six spectral scans were performed in the mid-range (MIR) region, from 4000 cm^−1^ to 650 cm^−1^, to define the characteristic peaks of the material.

## 3. Results

### 3.1. Tests Under Atmospheric Conditions

**Figure 5:**
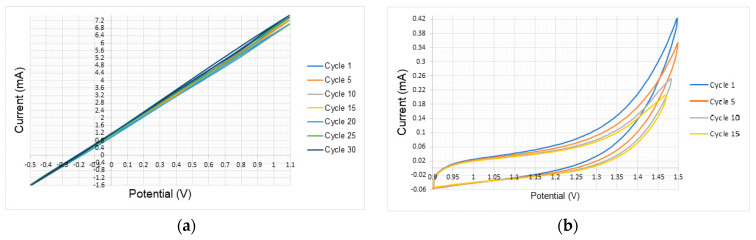

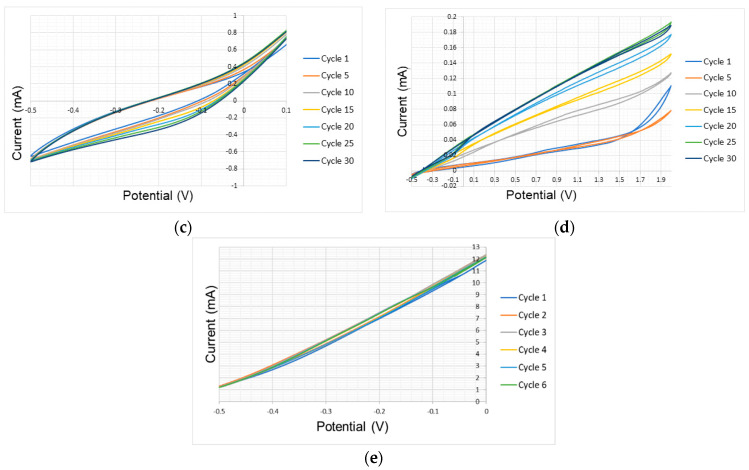
Experimental voltammograms under atmospheric conditions: (**a**) copper, (**b**) 316 stainless steel, (**c**) tin, (**d**) aluminum, (**e**) zinc.

**Figure 6:**
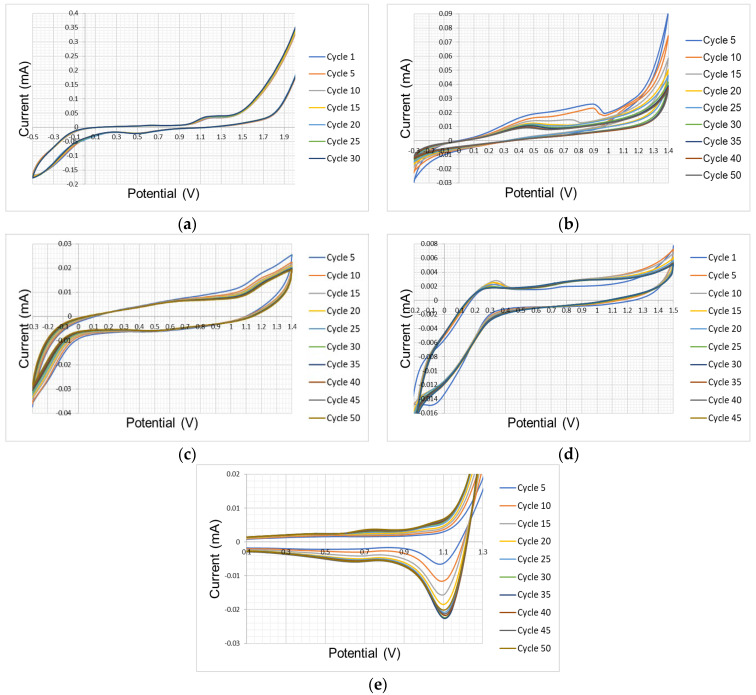
Experimental voltammograms under atmospheric conditions: (**a**) platinum, (**b**) nickel, (**c**) glassy carbon, (**d**) FTO, (**e**) gold.

Figure 5 and Figure 6 shows the experimental voltammograms under atmospheric conditions for each of the test materials.

The voltammogram with the copper electrode (Figure 5a) showed linear behavior, corresponding to its normal oxidation process (release of copper molecules from this material into the solution). Since no oxidation peaks were observed, no polymer formation (growth of P3HT on the metal surface) was reported when using this material. It is worth mentioning that the copper electrode contaminated the solution and negatively affected the experimental process. Therefore, the solution was discarded and replaced with a fresh one before continuing.

Unlike the copper, the voltammograms recorded with 316 stainless steel and tin electrodes (Figure 5b,c), a capacitive response and an oxidation process in the anodic region is observed. In contrast, with the aluminum electrode (Figure 5d), the behavior is semi-linear, with a progressive increase in the slope with the number of cycles. In these plots, the oxidation peaks were not observed indicating the negligible formation of the polymer on the electrode surface.

In the voltammogram with the zinc electrode (Figure 5e), as with the copper electrode, a linear behavior without oxidation peaks is observed, indicating a normal oxidation process without polymer formation (release of zinc molecules, but without growth of P3HT on the metal surface). It is worth noting that the zinc electrode, being a highly reducing material, could not be subjected to a potential level above 0 V to induce the formation of a polymer layer. Furthermore, only six cycles (from −0.5 to 0 V) could be performed before the equipment became overloaded.

In the presence of platinum (Figure 6a), oxidation peaks can be seen at 1.15 V after each cycle, indicating the growth of the P3HT. However, this oxidation peak is very small, suggesting a minor polymerization. Conversely, with a nickel electrode (Figure 6b) oxidation peak at −0.25 V and 0.7 V were observed for each cycle, indicating a growth of the P3HT polymer at atmospheric conditions on this material.

In the voltammogram obtained with the glassy carbon electrode (Figure 6c), a characteristic and prominent oxidation peak was observed at a constant potential of 0.47 V and 0.7 V after each cycle. This material could be suitable for the synthesis of P3HT. In the same manner, a characteristic and prominent oxidation peak was observed at a potential of 0.35 V on the FTO electrode (Figure 6d) related with the synthesis of the P3HT.

In the voltammogram of the gold electrode (Figure 6e), characteristic and outstanding oxidation peaks are observed at 0.73 and 1.1 V, related with the formation of the polymer. It is presumed that this is the best behavior of all the materials tested under atmospheric conditions.

**Figure 7:**
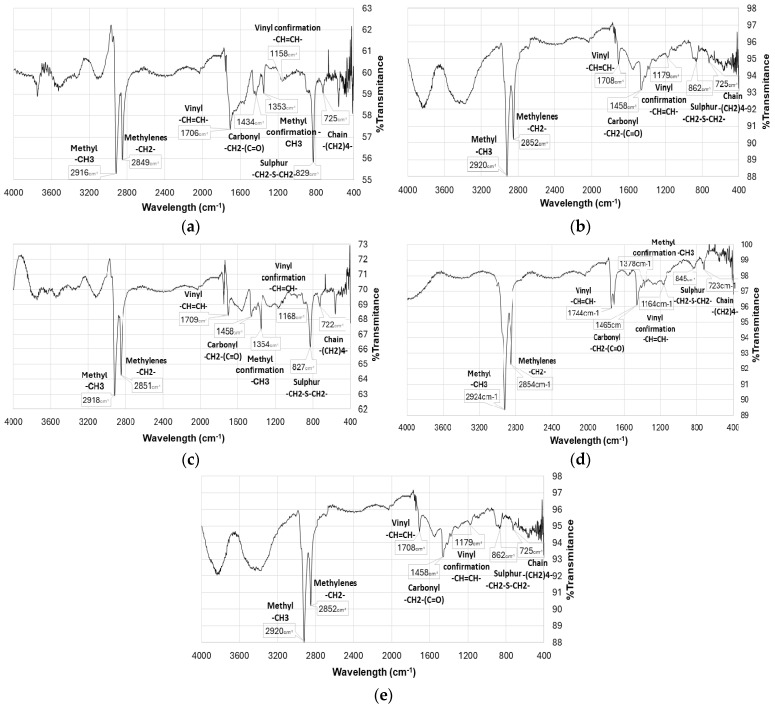
MIR spectrum of P3HT deposited on the surface of the test electrodes under atmospheric conditions: (**a**) platinum, (**b**) nickel, (**c**) glassy carbon, (**d**) FTO, (**e**) gold.

To confirm the presence of P3HT deposited on the electrodes, a FT-IR characterization was performed in the range of 4000 cm^−1^ to 400 cm^−1^. The MIR spectra obtained at each electrode are shown in Figure 7.

The peaks of the characteristic MIR spectra of the functional groups of P3HT are shown, highlighting the characteristic bands around 2920 and 2850 cm^−1^, corresponding to the C-C and C-H absorption bands of methyl and methylene.

### 3.2. Tests in Inert Atmosphere (Controlled Conditions)

**Figure 8:**
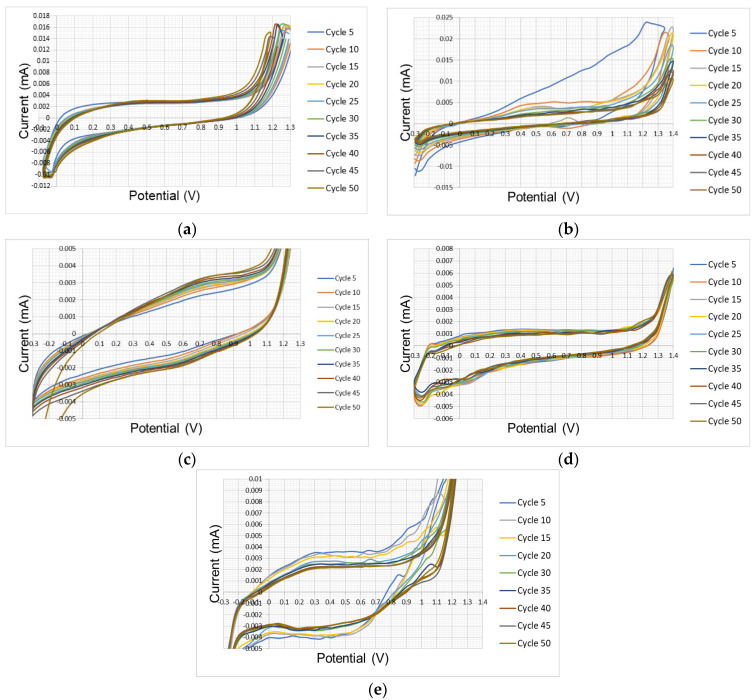
Experimental voltammograms under inert atmosphere: (**a**) platinum, (**b**) nickel, (**c**) glassy carbon, (**d**) FTO, (**e**) gold.

The electropolymerization process was performed under an inert atmosphere and the voltammograms are shown in Figure 8. This Figure showed greater differences compared to those obtained under atmospheric conditions (Figure 5 and Figure 6), with more pronounced oxidation peaks after each cycle.

In the voltammogram of the platinum electrode (Figure 8a), an oxidation peak at 0.5 V is observed related to the oxidation of the monomer and the growth of the polymer on this electrode. As was mentioned before, the current is minor compared to other metals, likely due to a minor thickness of the film.

Similarly, for the nickel electrode (Figure 8b), an oxidation peak at 0.5 V is evident for each cycle; indicating, the polymer formation on the surface The oxidation of the monomer is also observed for the glassy carbon electrode (Figure 8c) at 0.55 and 0.8 V, denoting a clear growth of the polymer.

Despite the noise response with FTO electrode (Figure 8d), a slight oxidation peak is observed at 0.35 V; meanwhile, in the voltammogram with gold electrode (Figure 8e), an oxidation peak is observed at 0.3 V and 0.45 V. It can be observed that the currents are larger compared with those recorded under atmospheric conditions (Figure 6e), suggesting a major polymerization on the electrodes. It is important to note that the film growth on the electrode surface was also observed with the naked eye.

**Figure 9:**
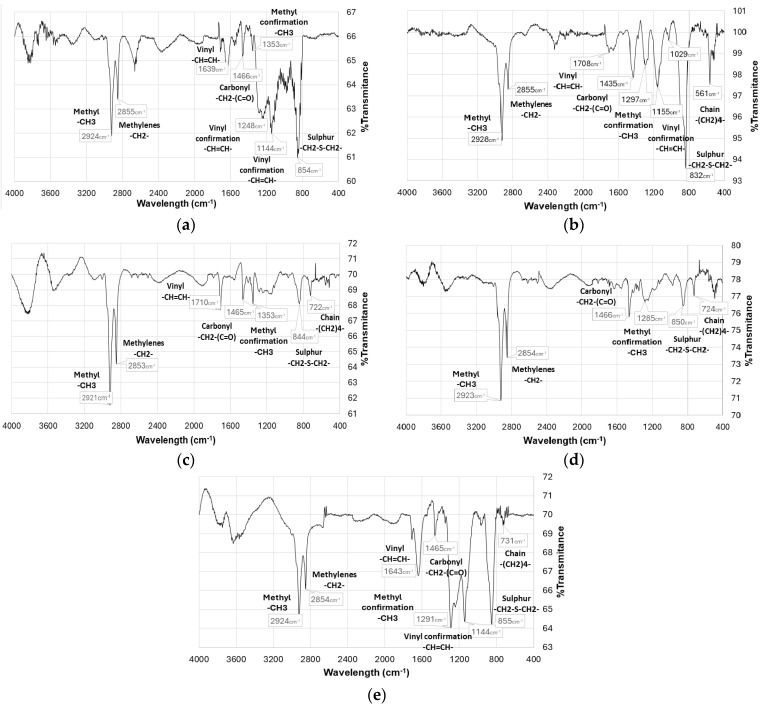
MIR spectrum of P3HT deposited on electrode under inert atmosphere: (**a**) platinum, (**b**) nickel, (**c**) glassy carbon, (**d**) FTO, (**e**) gold.

To confirm the presence of P3HT deposited on these electrodes, the electrode surface was characterized by FT-IR. The MIR spectra are shown on Figure 9. Similarities with those obtained under atmospheric conditions are observed, showing narrow bands at 2920 cm^−1^, 2850 cm^−1^, 1706 cm^−1^, 1430 cm^−1^, 829 cm^−1^ and 725 cm^−1^.

In the article by Kazutoshi Tanaka et al. [28], the synthesis of polythiophene (PT) using nickel, gold and platinum, among other materials, is described. These findings corroborate the results obtained; poly(3-hexylthiophene) (P3HT), being a derivative of polythiophene, allows synthesis using the same materials.

In the work by Juárez Fabiola [14], poly(3-hexylthiophene) was synthesized by chemical oxidation using FeCl_3_ as an oxidizing agent, chloroform as a solvent, and eliminating water; nitrogen gas was used for 24 h for its purification. However, during the experimentation in an inert atmosphere, the bubbling of nitrogen in the solution promotes volatility of the solvent, so prolonged bubbling is not advisable since it can cause total or partial evaporation of the solvent.

Table 1 shows a summary of the results for each material, indicating the range of applied potential, where the oxidation peak occurs and the current range in which the polymer growth on the surface.

## 4. Conclusions

The electropolymerization of poly(3-hexylthiophene) can be carried out using cyclic voltammetry, with the appropriate selection of materials and reagents for its production. It represents an environmentally friendly alternative for obtaining semiconductor polymers, compared to conventional methods that use free radical initiators, which are harmful to human health.

The proposed method used electrodes made of copper, tin, zinc, 316 stainless steel, aluminum, nickel, glassy carbon, gold, platinum and fluorine-doped tin oxide (FTO); the electrode working area was defined based on the electrode dimensions (length, width and thickness).

Tetrahydrofuran was selected to prepare the solvent solution because of its structural affinity with 3-hexylthiophene. Acetonitrile was chosen as a co-solvent because it does not interfere with tetrahydrofuran, which might otherwise interfere with the reaction. It also has affinity with the pendant groups present in 3-hexylthiophene. Lithium hexafluorophosphate has been shown to have moderate solubility in acetonitrile.

Experimental results, under atmospheric conditions with copper, 316 stainless steel, tin, aluminum or zinc, suggest that none of these materials are suitable for P3HT production. In contrast, platinum, nickel, glassy carbon, FTO and gold electrodes, visually showed growth on its surface, indicating possible polymer deposition.

Under controlled conditions, a significant impact on the growth of the polymer film on the working electrode was observed. Improvements in the coating of the FTO electrode and the nickel electrode were achieved in the presence of nitrogen, with a concentration of 0.01 M 3-hexylthiophene and 0.01 M lithium hexafluorophosphate under both conditions.

The voltammograms resulting from tests performed in the presence of nitrogen (under controlled conditions) show an increase in the applied current range, suggesting that the polymer film growth is greater compared to tests performed in the absence of nitrogen. Despite the turbulence shown in the graphs, caused by the movement of the solution due to the injection of gaseous nitrogen, oxidation peaks related to the growth of the polymer layers on the electrode are observed.

Overall, the experimental results obtained revealed that, with the exception of the zinc electrode, a highly reducing material, it does not allow for the application of a wider potential range. The size and useful area of the working electrode is inversely proportional to the potential range that can be applied to the electrodes; that is, the larger the size and useful working area, the narrower the potential range, and vice versa. Therefore, a wider working potential range was obtained for the gold, platinum, nickel and glassy carbon electrodes.

MIR spectra obtained in tests conducted in the presence of nitrogen generate more pronounced and defined absorption peaks than those observed in the absence of nitrogen, due to greater growth on the working electrode surface. These observations demonstrate the presence of functional groups belonging to the polymer chain of poly(3-hexylthiophene) (P3HT).

The proposed method presents an alternative application of electropolymerization for the synthesis of poly(3-hexylthiophene) (P3HT). This study is relevant given that P3HT is widely used in polymer-based optoelectronic applications and explores efficient synthesis routes of interest to the scientific community. This is especially true considering that, unlike conventional solutions, it allows the synthesis of P3HT without the generation of free radicals, which are harmful to the environment and the human body.

Future work: To promote improvements in the synthesis process and obtain a thicker coating of this material, it is recommended to test different work area configurations applying different potential ranges, as well as different supporting electrolytes (belonging to ionic liquids), with a higher concentration of the monomer used, e.g., lithium hexafluorophosphate (LiPF_6_) as the supporting electrolyte and carbonate and/or dimethyl carbonate as cosolvent. Information on the electronic structure, its potential and its suitability for different applications could be verified by measuring the optical band gap using UV-VIS spectroscopy, including information on the HOMO-LUMO energy levels from the obtained results.

## Figures and Tables

**Table 1:** Summary of voltammogram results.

Atmospheric Conditions Test Results				
Electrode	Potential Range (V)	Oxidation Peaks (V)	Current Range (mA)	Polymer Growth
Copper	−0.5–1.1	-	−1.6–7.4	-
Zinc	−0.5–0	-	1–12	
Tin	−0.5–0.1	-	−0.7–0.8	
316 Stainless Steel	0.9–1.5	-	−0.06–0.38	
Aluminum	−0.5–2	-	−0.02–0.2	
Nickel	−0.3–1.4	0.44	−0.03–0.09	**✔**
Glassy Carbon	−0.3–1.4	0.47 and 0.7	−0.04–0.02	
Gold	0–1.3	0.74 and 1.12	−0.023–0.02	
Platinum	−0.5–2	0.5 and 1.2	−0.17–0.3	
FTO	−0.2–1.5	0.33	−0.02–0.008	
**Test results in inert atmosphere**				
**Electrode**	**Potential Range (V)**	**Oxidation Peaks (V)**	**Current Range (mA)**	**Polymer growth**
Nickel	−0.3–1.4	0.5	−0.01–0.025	**✔**
FTO	−0.3–1.4	0.35	−0.005–0.006	
Platinum	−0.3–1.4	0.5	−0.012–0.018	
Glassy Carbon	−0.3–1.4	0.55 and 0.8	−0.005–0.005	
Gold	−0.3–1.3	0.3 and 0.45	−0.005–0.01	

## Data Availability

The original contributions presented in this study are included in the article. Further inquiries can be directed to the corresponding authors.

## Abbreviations

The following abbreviations are used in this manuscript:

P3HT	Poly(3-hexylthiophene)
FT-IR	Fourier transform infrared spectroscopy
ICPs	intrinsically conducting organic polymers
PPP	poly(p-phenylene)
PA	polyacetylene
PT	polythiophene
PPV	poly(p-phenylene-vinylene)
PANI	polyaniline
PPy	polypyrrole
P3ATs	poly(3-alkylthiophene)
FETs	Field Effect Transmiter
OLEDs	Light-Emitting Diodes
LiPF6	lithium hexafluorophosphate
FTO	fluorine-doped tin oxide
3HT	3-hexylthioene
AcN	acetonitrile
THF	Tetrahydrifuran
WE	working electrode
CE	counter electrode
N_2_	nitrogen flow
ATR	Atenuated Total Reflectance
MIR	mid-range
LiPF_6_	Heaflourofosfate
